# Belgian rare diseases plan in clinical pathology: identification of key biochemical diagnostic tests and establishment of reference laboratories and financing conditions

**DOI:** 10.1186/s13023-021-01728-1

**Published:** 2021-02-17

**Authors:** Nathalie M. Vandevelde, Pieter Vermeersch, Katrien M. J. Devreese, Marie-Françoise Vincent, Béatrice Gulbis, François Eyskens, François Boemer, André Gothot, Viviane O. Van Hoof, Carolien Bonroy, Hedwig Stepman, Geert A. Martens, Xavier Bossuyt, Laurence Roosens, Julie Smet, Hilde Laeremans, Ilse Weets, Jean-Marc Minon, Kris Vernelen, Wim Coucke, Advisory Board of the Action 1 of the Belgian National Plan for Rare Diseases, Bernard Debbaut, Katrien M. J. Devreese, François Eyskens, Céline Franken, Yves Gillerot, Béatrice Gulbis, Chantal Mathy, Marc Moens, Nathalie M. Vandevelde, Philippe Van de Walle, Pieter Vermeersch, Marie-Françoise Vincent

**Affiliations:** 1 Department of Quality of Laboratories, Sciensano, Rue Juliette Wytsmanstraat 14, 1050 Brussels, Belgium; 2 Rare Diseases Working Group, Belgian National Commission on Clinical Pathology, Brussels, Belgium; 3 Department of Laboratory Medicine, UZ Leuven, Leuven, Belgium; 4 Department of Cardiovascular Sciences, University of Leuven, Leuven, Belgium; 5 Department of Laboratory Medicine, Ghent University Hospital, Ghent, Belgium; 6 Department of Laboratory Medicine, Cliniques Universitaires Saint-Luc and Université Catholique de Louvain, Brussels, Belgium; 7 Belgian Fund Rare Diseases and Orphan Drugs, Brussels, Belgium; 8 Clinical Pathology, LHUB-ULB, Université Libre de Bruxelles, Brussels, Belgium; 9 Center of Inherited Metabolic Diseases, Antwerp University Hospital, Edegem, Belgium; 10 Department of Metabolic Disorders in Children, Antwerp University Hospital, Edegem, Belgium; 11 Observatory of Chronic Diseases, National Institute for Health and Disability Insurance (INAMI-RIZIV), Brussels, Belgium; 12 Biochemical Genetics Lab, Department of Human Genetics, CHU of Liege, University of Liege, Liège, Belgium; 13 Department of Laboratory Haematology and Immuno-Haematology, CHU Liège, Liège, Belgium; 14 Department of Clinical Chemistry, Antwerp University Hospital, Edegem, Belgium; 15 VUB Metabolomics Platform, Vrije Universiteit Brussel, Brussels, Belgium; 16 Laboratory for Molecular Diagnostics, AZ Delta Roeselare, Roeselare, Belgium; 17 Laboratory for TDM and Toxicology, University Hospital Antwerp, Edegem, Belgium; 18 Laboratory of Pediatric Research, Free University of Brussels, Brussels, Belgium; 19 Department of Clinical Chemistry and Radio-Immunology, University Hospital Brussels, Brussels, Belgium; 20 Laboratory and Department of Blood Transfusion, CHR de la Citadelle, Liège, Belgium

**Keywords:** Rare diseases, Clinical pathology, Financing, Reference laboratories, Reimbursement codes, Expertise

## Abstract

**Background:**

One objective of the Belgian Rare Diseases plan is to improve patients’ management using phenotypic tests and, more specifically, the access to those tests by identifying the biochemical analyses used for rare diseases, developing new financing conditions and establishing reference laboratories.

**Methods:**

A feasibility study was performed from May 2015 until August 2016 in order to select the financeable biochemical analyses, and, among them, those that should be performed by reference laboratories. This selection was based on an inventory of analyses used for rare diseases and a survey addressed to the Belgian laboratories of clinical pathology (investigating the annual analytical costs, volumes, turnaround times and the tests unavailable in Belgium and outsourced abroad). A proposal of financeable analyses, financing modalities, reference laboratories’ scope and budget estimation was developed and submitted to the Belgian healthcare authorities. After its approval in December 2016, the implementation phase took place from January 2017 until December 2019.

**Results:**

In 2019, new reimbursement conditions have been published for 46 analyses and eighteen reference laboratories have been recognized. Collaborations have also been developed with 5 foreign laboratories in order to organize the outsourcing and financing of 9 analyses unavailable in Belgium.

**Conclusions:**

In the context of clinical pathology and rare diseases, this initiative enabled to identify unreimbursed analyses and to meet the most crucial financial needs. It also contributed to improve patients’ management by establishing Belgian reference laboratories and foreign referral laboratories for highly-specific analyses and a permanent surveillance, quality and financing framework for those tests.

**Supplementary Information:**

The online version contains supplementary material available at 10.1186/s13023-021-01728-1.

## Introduction

In Europe, rare diseases are defined as disorders affecting less than 1 patient in 2.000 individuals [1, 2]. To this date, approximately 8.000 rare diseases have been identified [2–5]. Their diagnosis is often delayed because of the diversity, complexity and rarity of these disorders and the lack of access to specialized diagnostic tools [2, 4–6]. A recent survey of patients with mitochondrial disorders, for example, showed that most of them consult five or more clinicians and received at least one incorrect diagnosis before the establishment of the final diagnosis [7]. In 2009, the Council of the European Union published recommendations addressed to the Member States in order to encourage them to improve the access of patients with rare diseases to high-quality diagnosis, care, treatment, social support and information [2, 8, 9].

One of the deliverables of the Belgian action plan for rare diseases, published in December 2013, is to improve the patients’ diagnosis and follow-up [10]. Because most of rare diseases have a genetic origin, the Belgian centers of human genetics were firstly involved in the process, followed by a specific action focused on biochemical laboratory tests performed by medical laboratories. Indeed, genetic and biochemical tests are highly complementary in order to investigate and follow rare diseases’ mechanisms, the underlying biochemistry, cellular pathways and response to treatments [11].

A critical analysis of the Belgian situation in 2015 identified three factors limiting the patients’ access to specialized biochemical laboratory tests: (1) lack of reimbursement for a number of specialized tests within the framework of the public health system, (2) absence of reference laboratories with scientific and technical expertise for complex tests, and (3) need for an official referral framework for outsourcing tests unavailable in Belgium to foreign laboratories.

To address these shortcomings, the Belgian Institute for Health (Sciensano) was mandated by the Belgian National Institute for Health and Disability Insurance (RIZIV-INAMI) to (1) review the reimbursement of biochemical tests prescribed in Belgium in the context of rare diseases, and (2) organize the selection, recognition and financing of Belgian reference laboratories (RLs) and foreign referral laboratories for selected biochemical tests. A task force was established by Sciensano with clinical pathologists and clinicians.

This paper describes the approach used to identify and prioritize the biochemical analyses that should be covered by the Belgium public health system, either through traditional reimbursement of clinical pathology tests or through the development of a new system of RLs and foreign referral laboratories, as well as RLs’ selection procedure and duties.

## Materials and methods

### Data collection

In May and June 2015, Sciensano performed a screening of the European expert centres and medical laboratories providing diagnostic tests used in the context of rare diseases published on Orphanet [12], and of peer-reviewed scientific publications describing the use and validation of biochemical tests used for rare diseases.

The inventoried analyses were classified based on their respective domains (clinical chemistry, endocrinology, haematology, coagulation and hemostasis, immunology and non-infectious serology, toxicology, therapeutic monitoring) and, when necessary, subclassified according to biological matrices (blood, plasma, serum, dry blood spots, urine, urinary stones, feces, cerebrospinal fluid, saliva, amniotic fluid) and/or chemical classes of metabolites/proteins (amino acids and derivates, pterins, organic acids, steroid acids, purines/pyrimidines, sugars, lipids and acylcarnitines, porphyrins, vitamins, different proteins classes [e.g. transcobalamines, chemokines, iron-binding blood plasma glycoproteins, lectins, (apo-) lipoproteins, immunoglobulins, serum free light chains], cytoplasmic enzymes, lysosomal enzymes, mitochondrial enzymes, inhibitors [e.g. Alfa-1-antitrypsin], regulators [e.g. 14-3-3 proteins], complement control proteins [e.g. Factor H], cofactors [e.g. molybdenum cofactor], and other metabolites and components [e.g. α-aminoadipic semialdehyde, trimethylamine, sulfites, etc.]). Of note, one metabolite assessed in two different matrices, was inventoried as two different analyses.

Subsequently, a survey was sent in July 2015 to the Belgian laboratories of clinical pathology performing those analyses.

For each test, these laboratories had to provide the following information:

- The annual number of tests performed;
- The rare disease(s) for which the test is performed;
- For analyses not performed in-house, the name of the external laboratory to which the analysis is outsourced and whether the laboratory wanted to develop it in-house by 2020;
- The average turnaround time (TAT). The TAT was defined as the time between the reception of the sample by the laboratory and the moment the result is reported (in-house or by a foreign laboratory) [13].

The survey was accompanied by a cover letter specifying the context and objectives of the study, and ensuring that collected data would be stored and treated in complete confidentiality and anonymity. Participants had over two months to complete it and were free to add additional tests of clinical pathology if they were not yet included in the list. All answers were then collected and put together by Sciensano.

### Analyses prioritization

In November 2015, a “Rare Diseases Working Group (RDWG)”, composed of clinical pathologists with a particular expertise in rare diseases and one Sciensano scientific moderator, was set-up by the Belgian Commission on Clinical Pathology.

Between December 2015 until June 2016, the RDWG met 2 to 5 times per month in order to discuss the usefulness, clinical relevance, limitations and costs of the analyses for which information was collected during the survey. The goal was to evaluate the feasibility of their financing and identify analyses which should be prioritized for reimbursement. Particular attention was paid to tests for which there was disagreement about the clinical utility. The goal was to sort out whether discrepancies of opinions were due to evidence-based disagreements over the use of the analysis in clinical practice or to differences in laboratories’ functioning.

### Funding modalities

The INAMI-RIZIV proposed three possible financing modalities: (a) nomenclature reimbursement codes, (b) recognition and financing of RLs, or (c) financing of the outsourcing of analyses unavailable in Belgium to foreign laboratories. At the request of Sciensano, the RDWG defined for each test the financing modality and the total the annual budget that should be allocated to each type of financing modality.

In August 2016, Sciensano submitted a proposal of priority analyses that should be funded and selection criteria for the Belgian RLs to the RIZIV-INAMI. The description of each analysis was accompanied by an annual budget estimate based on the analytical costs and type of financing modality proposed by the RDWG. The proposal of priority analyses and required annual budget was approved by the INAMI-RIZIV in December 2016.

### Implementation phase

The approved proposal was implemented by Sciensano and the RDWG from January 2017 until August 2018. This consisted of (1) writing the nomenclature reimbursement codes (including reimbursement modalities) and publishing them in a Belgian royal decree (2) selecting the Belgian RLs, and (3) formalizing official collaborations with some foreign laboratories. The INAMI-RIZIV evaluated this proposal from October 2018 until December 2019.

### Statistical analyses

One-way analysis of variance (ANOVA) was made with GraphPad Prism 8.2.0 (GraphPad Software, San Diego, CA).

## Results

**Fig. 1:**
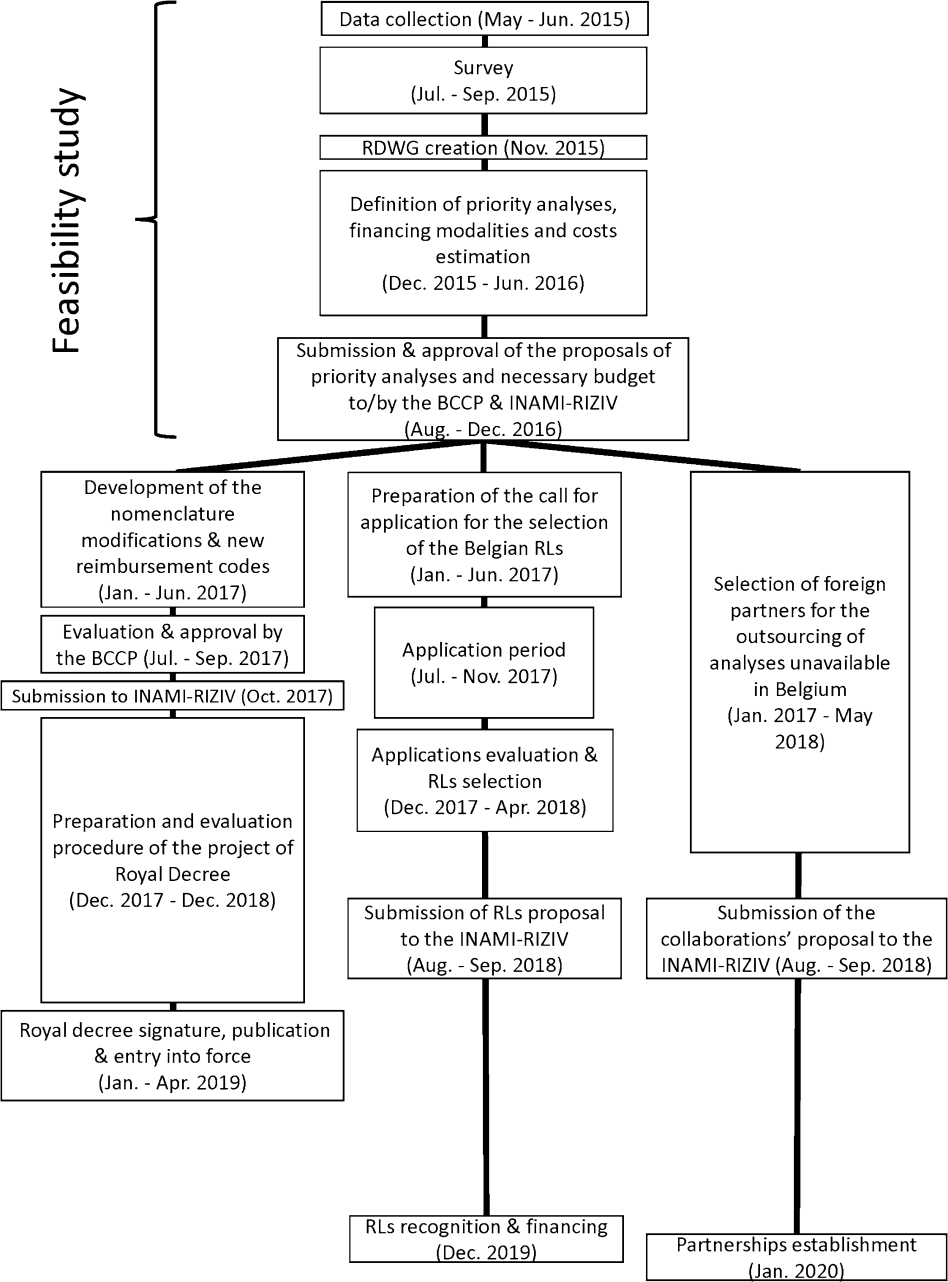
Illustration of the different steps of the study. *BCCP* Belgian Commission on Clinical Pathology, *INAMI-RIZIV* National Institute for Health and Disability Insurance, *RD* Royal Decree, *RDWG* Rare Diseases Working Group, *RLs* Reference laboratories

Figure 1 illustrates the study outline.

### Analyses inventory

Unreimbursed analyses of clinical pathology used in the context of rare diseases were identified based on the literature search and survey that was sent to the 17 Belgian laboratories of clinical pathology performing these analyses (8 university laboratories and 9 non-university laboratories).

### Survey results

All laboratories completed the survey. When necessary, they added analyses that they perform and that were not included in the initial survey. The survey enabled Sciensano to identify 483 analyses of clinical pathology used in the context of rare diseases. Of these tests, 163 (34%) were unreimbursed in July 2015 and, therefore covered by the Belgian public healthcare system. In this case, the patient’s personal share varied between 0 and 20 euros per laboratory test request.

### Selection of priority analyses

Among the unreimbursed analyses, the RDWG selected priority tests based on the survey answers and, more specifically, on (1) the lack of existing reimbursed tests in the Belgian nomenclature for a rare disorder, (2) the absence of existing proposals for reimbursement already submitted to the Belgian healthcare authorities, (3) the clinical utility of the test (showing higher medical and/or analytical benefits compared to other existing techniques; some obsolete tests were replaced as part of the exercise), (4) the high specificity of the analyses for rare diseases (analyses widely performed in other contexts than the diagnosis or follow-up of rare diseases patients, were not included in the list of priority analyses).

**Fig. 2:**
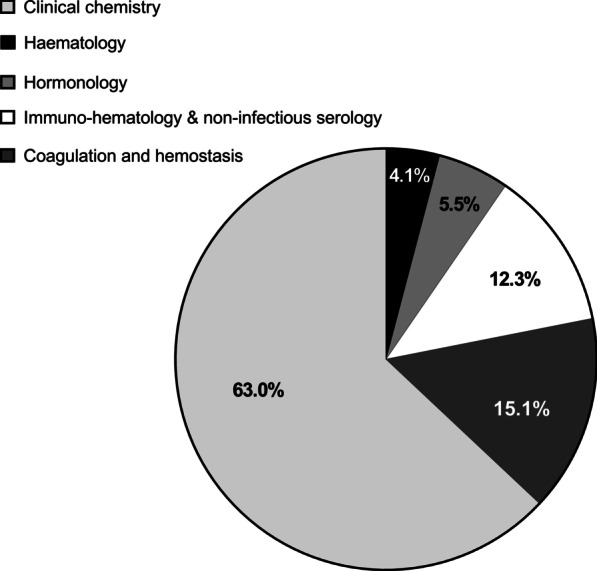
Repartition of the selected financeable priority analyses among their respective domains of clinical pathology

Using those criteria, 73 priority analyses were selected for coverage in the following fields: clinical chemistry (46), coagulation and hemostasis (11), immuno-haematology and non-infectious serology (9), hormonology (4), and haematology (3) (cf. Fig. 2). Among them, 64 analyses were performed in Belgium and 9 outsourced abroad. Moreover, 62 of the 64 analyses performed in Belgium (97%) were only performed in university hospital laboratories. A great disparity was observed among the annual volumes (from 1 to more than 5000 tests per year), as well as TAT (ranging from 40 h up to 2 months).

### Definition of financing modalities

Table 1 summarizes the characteristics of the three types of financing modalities.

**Table 1:** Financing modalities characteristics

Financing modalities	Nomenclature reimbursment codes	Reference laboratories (RLs)	Conventions with foreign laboratories
Type of medical analyses	Analyses of clinical pathology		
Analyses’ availability	Available in Belgium		Not available in Belgium
Annual volume	High	Moderate to low	Low
Laboratories who can benefit from the financing	All Belgian laboratories of clinical pathology	Only Belgian laboratories of clinical pathology recognized as RLs	Specific foreign laboratories of clinical pathology
Laboratories’ selection procedure	None	Belgian call for application By Sciensano Every 5 years	By the RDWG of the BCCP and Sciensano Every year
Components of the financing	Performance of the analysis	Analytical costs Accreditation Quality controls Specific administrative costs	Analytical costs Shipment costs
Financing source	INAMI-RIZIV (through a budget envelop specifically dedicated to the Action 1 of the Belgian plan for rare diseases)		
Follow-up of laboratories’ activities and quality of the analyses	By the RDWG of the BCCP and Sciensano		
Annual evaluation of the necessary budget	By Sciensano and the INAMI-RIZIV Insurance Committee		

*BCCP* Belgian Commission on Clinical Pathology, *INAMI-RIZIV* National Institute for Health and Disability Insurance, *RDWG* Rare Diseases Working Group, *RLs* reference laboratories

For analyses performed in Belgium, the choice of developing a reimbursement nomenclature code versus selecting a Belgian reference laboratory (RL) was based on the degree of centralization of the performance of the test (performed by a limited versus larger number of laboratories) and the required level of medico-scientific expertise and/or specialized infrastructure.

**Fig. 3:**
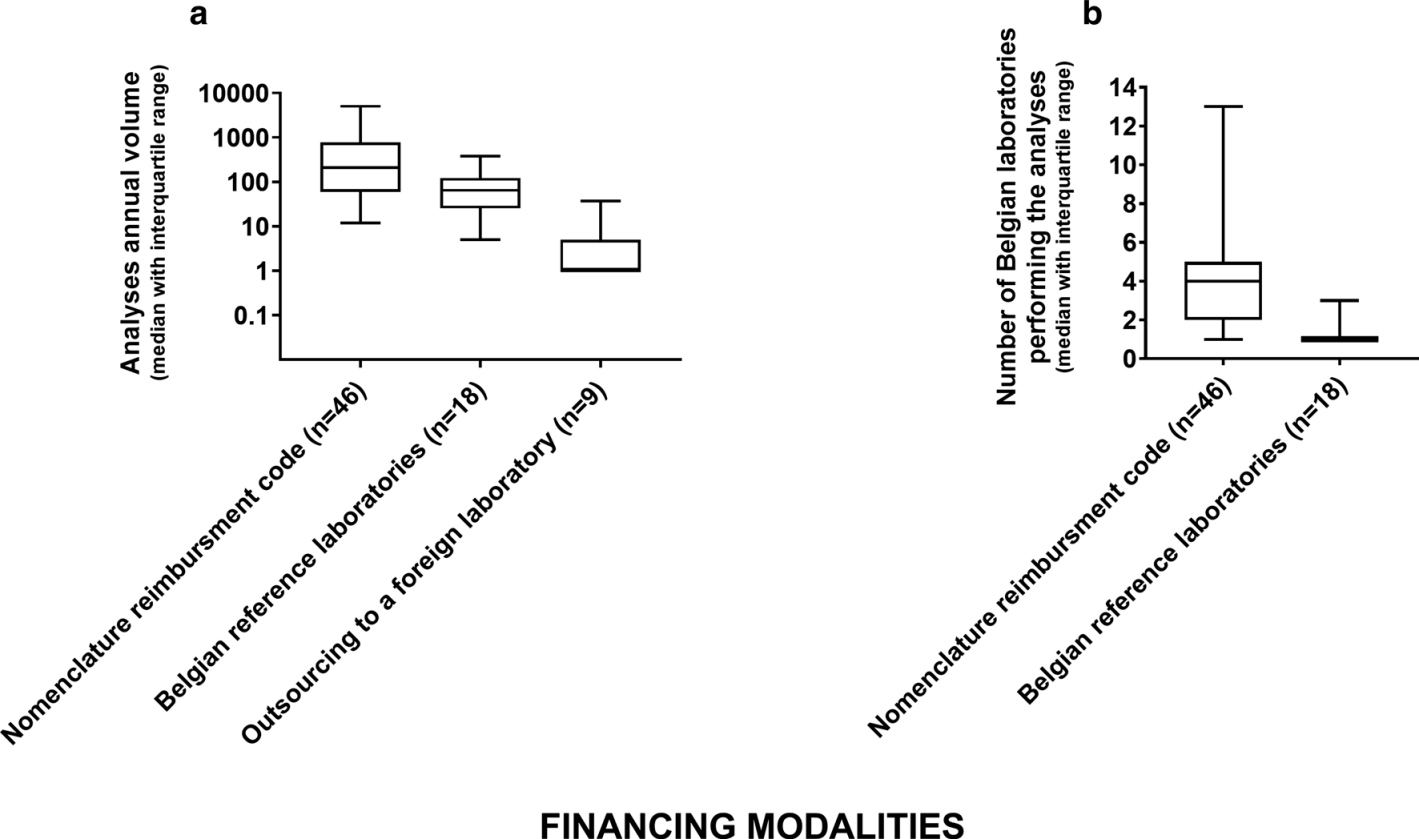
Volumes reported in 2016 for the 73 priority analyses categorized according to their financing modality

The development of nomenclature codes or modification of existing codes was favored for analyses performed by at least 3 Belgian laboratories for clinical pathology. The selection of RLs on the other hand was preferred for analyses characterized by a low annual volume, requiring specific infrastructure and/or scientific expertise, and performed by 1 to 3 Belgian laboratories (cf. Fig. 3a, b). Finally, the development of formal collaborations with foreign laboratories was proposed to cover analytical and shipment costs for low volume analyses unavailable in Belgium and thereby outsourced abroad.

### Development of reimbursement nomenclature codes

The Belgian reimbursement system for laboratory tests combines a fee for service per test and a flat rate which varies in function of the tests requested. The INAMI-RIZIV is responsible for establishing the flat rates, reimbursement fee per test and reimbursement rules for test (e.g. maximum one per year, only reimbursed in patients with a specific disorder, etc.), as well as for organizing, managing and supervising its correct application [14–16].

A reimbursement code for a clinical pathology test contains the (1) name of the test including possible additional technical requirements, (2) domain of clinical pathology to which the test belongs, (3) biological matrix, (4) theoretical reimbursement tariff represented by a ‘B-value’, (5) maximal frequency of reimbursement (e.g. maximum 1 test/day) and, if applicable, (6) diagnostic and/or cumulation rules. Of note, the theoretical tariff is calculated by multiplying the analysis’ B-value by the B-coefficient which is regularly adapted by the INAMI-RIZIV (current value: B = 0032012 since 01/01/2020 [17]). For instance, a B-value of 1000 corresponds to a theoretical tariff of approximately 32€ (including reagents/materials, personnel, quality controls costs). The fee of service and flat rate are calculated based on the theoretical tariffs of the requested tests.

Diagnostic rules give an accurate description of the context in which the test can be reimbursed (e.g. specific patient population, clinical symptoms) while cumulation rules define which tests cannot be combined for reimbursement.

The costs of the medical services (including medical laboratory analyses) that are not included in the Belgian reimbursement system are invoiced to the patients. By contrast, 25% of the costs of medical services with a nomenclature code are reimbursed by the INAMI-RIZIV. The rest is mainly charged to the health insurance of the patient. In some cases (not applicable to patients with chronic diseases), the patient pays a small amount that represents the difference between the cost of the medical service and the interventions of the INAMI-RIZIV and health insurance.

Between March and July 2016, the RDWG proposed nomenclature codes for all the priority tests selected for a reimbursement. Based on the B-values and number of tests performed reported in the survey, the total annual budget was calculated and submitted in August 2016 to the INAMI-RIZIV. After the budget approval, diagnostic and cumulation rules were developed by the RDWG between January and June 2017. The proposal for new reimbursement codes was approved by the Belgian Commission on Clinical Pathology in September 2017 and submitted to the INAMI-RIZIV in October 2017. After evaluation and approval by different INAMI-RIZIV and external bodies (i.e. Technical Medical Council, Insurance Committee, national medical-mutualistic commission), a royal decree project was prepared and submitted to the Belgian Budget ministry and Healthcare and Social Affairs ministry. The royal decree formalizing the modification of 4 existing nomenclature reimbursement codes and the creation of 42 new codes was published on 3 February 2019 and came into effect on 1 April 2019 [18]. Table 2 contains the list of the 42 new nomenclature codes.

**Table 2:** List of the 42 new reimbursement nomenclature codes [18]

Domains of clinical pathology	Biological matrices	Names of the nomenclature codes
Clinical chemistry	Blood	Separated identification and assessment of C22–C26 fatty acids, phytanic acid and pristanic acid by mass spectrometry
		Methylmalonic acid and succinylacetone assessment
		Pipecolic acid assessment
		Guanidinoacetate and creatine assessment
		Measurement of alpha-d-galactose 1-phosphate (Gal-1-P) in erythrocytes
		Separated assessment of cholestanol, 7-dehydrocholesterol and 8-dehydrocholesterol
		Separated assessment of desmosterol, lathosterol, campesterol, sitosterol and 27-hydroxycholesterol
		Identification of asialotransferrin, monosialotransferrin, and asialotransferrin
		Determination of the Alpha-1-proteinase inhibitor (= alpha-1-antitrypsin) phenotype
	Serum/plasma	Organic acids assessment
	Urine	Guanidinoacetate and creatine assessment
		Separated assessment of polyols after fractionation
		Separated assessment of mono- and disaccharides after fractionation
		Separated assessment of polyols, monosaccharides and disaccharides after fractionation
	Cerebrospinal fluid	Specific mass spectrometric identification and dosage of organic acids
		Specific assessment of amino acids by LC–MS/MS after derivatization and fractionation
		Guanidinoacetate and creatine assessment
	Stool	Measurement of pancreatic Elastase 1
	Amniotic fluid	Separated assessment of 7-dehydrocholesterol and 8-dehydrocholesterol
Hormonology	Blood	Assessment of fibroblast growth factor 23
		Assessment of pregnenolone
		Assessment of 17-hydroxypregnenolone
		Proinsulin measurement
	Saliva	Cortisol measurement
Haematology	Blood	Eosin-5′-maleimide (EMA) binding test
Coagulation and hemostasis	Plasma	Alpha 2-Antiplasmin measurement
		Functional analysis of von Willebrand factor-cleaving protease (ADAMTS13)
		Identification of the von Willebrand factor‐cleaving protease inhibitor
		Diagnosis of type 2N von Willebrand disease
		von Willebrand factor collagen-binding activity assay
		von Willebrand factor multimer analysis
		von Willebrand factor propeptide assay
		Detection of heparin-induced antibodies (= diagnosis of heparin induced thrombocytopenia [HIT])
		Functional analysis of HIT antibodies
		Platelet secretion assay
		Measurement of prekallikrein (Fletcher Factor) anticoagulant activity and of high-molecular-weight kininogen
Immuno-haematology and non-infectious serology	Serum	Detection of anti-podocytes antibodies in the context of membranous glomerulonephritis diagnosis
		Detection of anti-podocytes antibodies in the context of membranous glomerulonephritis follow-up
		Determination of anti-acetylcholine receptor antibodies
		Immunoassay identification of autoantibodies against bullous pemphigoid (BP) antigen 180, BP230 (bullous pemphigoid antigen 1), DSG1 (desmoglein 1), DSG3 (desmoglein 3), type VII collagen and envoplakin
		Follow-up of the production of autoantibodies against bullous pemphigoid (BP) antigen 180, BP230 (bullous pemphigoid antigen 1), DSG1 (desmoglein 1), DSG3 (desmoglein 3), type VII collagen and envoplakin by immunoassay

### Selection of the reference laboratories (RLs)

The selection criteria for the Belgian RLs are summarized in Table 3. Special attention was paid to the laboratories’ quality management system, its medico-scientific expertise (including the extent of its collaborations with external rare diseases experts), the education program addressed to the laboratory staff and medical prescribers, and whether the laboratory will be able to offer the test during at least 5 years.

**Table 3:** Illegibility criteria for the selection of the Belgian Reference laboratories

Main aspects	Criteria to be met	Evaluation procedure
Localisation, structure, organization and governance	Applicants are laboratories of clinical pathology located in Belgium and certified according to the Royal Decree of 3 December 1999 [19] Applicants provide the full contact details of the laboratory, its director, quality coordinator and of the clinical pathologist(s) responsible for the performance of the analysis for which they apply Applications in the form of a consortium have to be justified A clear description of the tasks performed by each laboratory composing the consortium must be provided	Documentation audit On-site visits for RL in the form of a consortium
Quality	Applicants have to join the following documents to the application file: Full content of the laboratory quality manual Description of the method and equipment used to perform the analysis (Standard Operating Procedure) Analysis validation file (including the reference values and bibliographic references used to validate the method and interpret analytical results) Description of the External Quality Assessment programs and/or ring tests related to the analysis for which they apply and to which they participate Copy of the laboratory accreditation certificate according to the norm ISO15189 [20]	Documentation audit On-site visits
Reporting of analytical results and communication skills	Applicants have to specify: The units used to report the results and if those units are SI units or not (if not, they also have to mention the reason) Their mean turnaround time (TAT)^a^ for the analysis for which they apply (expressed in days, with a standard deviation if desired) The link to their website where the prescribers can find the analytical TAT If they undertake to respect the TAT mentioned on their website/prescription forms If they undertake to be more flexible and reduce their TAT for urgent analyzes in order to guarantee the good management of the patient If they are able to report the results in at least one of the Belgian national languages and in English If they accept to publish the prescription forms in French, Dutch and English on their website If they accept that information related to their activities (no patients details/personal information) will be used to update and enrich the Belgian rare diseases registries	Documentation audit
Scientific and medical expertise	Applicants have to provide: Their annual volume of tests during the 5 last years for the analysis for which they apply Their list of scientific communications (peer-reviewed publications, posters, etc.) related to rare diseases management A short description of their projects in the domain of clinical pathology to which the analysis for whom they apply belongs (basic research projects, clinical research, epidemiological research, validation of new methods, acquisition of new equipment, etc.) Their affiliations to international (reference) networks with a short description of the (1) network spectrum of activities, (2) applicants duties/activities within the network, and (iii) frequency of applicants’ meetings/exchanges of information with other network members The names of the databases in which analytical results are recorded (patient’s medical record, laboratory database, Regional/national database, network database, etc.) A description of their participation to multidisciplinary meetings (frequency, medical disciplines [specializations] of the experts who attended the meetings, aspects discussed during the meetings [clinical cases, analytical results interpretation, communication between the laboratory and prescribers, development of guidelines/healthcare algorithms, etc.]) A description of the diagnosis and/or follow-up guidelines/algorithms that they may develop for some rare diseases A description of the training programs that they develop and of the targeted audience	Documentation audit
Activities sustainaibility	Candidates undertake to carry out the analysis for which they apply for at least 5 years in the case of a recognition as RL and confirm that they possess the necessary qualified personnel, equipment and infrastructure to carry out the analysis during this period of time	Documentation audit

*RL* reference laboratory, *SI* International System of Units, *TAT* Turnaround Time

^a^For analyses made in Belgium, the TAT corresponds to the time period between the reception of the sample by the laboratory and the moment at which the clinician receives the validated results; for analyses outsourced abroad, it corresponds to the time period between the test prescription and the moment at which this clinician receives the validated results

The call for application was prepared by Sciensano between January and June 2017 and included:

1. an introductory letter explaining the call’s context and objectives;
2. an explanatory document describing the applicants’ profile, RLs missions, application documents, selection procedure, and analyses for which applicants could apply for a recognition of expertise;
3. the application documents that must be completed in English (application form and agreement forms for the submission of the application signed by the laboratory director and clinical pathologist responsible for the performance of the test);
4. French and Dutch translations of the application documents provided to ensure an optimal comprehension of the application documents written in English.

The call for application was officially opened on 1 July 2017. The laboratories had five months to apply for one or several analyses. Moreover, the laboratories were free to apply together in the form of a consortium. In this case, the relevance of the consortium had to be justified in the application form.

The evaluation procedure took place between December 2017 and April 2018. All applications were reviewed by three independent experts (not linked to the Belgian laboratories of clinical pathology). This was performed through documentation audits of the application form, laboratory quality manual, accreditation certificate, validation file and standard operating procedure (SOP) for the performance of the analysis, peer-reviewed publications illustrating the laboratory scientific expertise and collaborations, guidelines, decision algorithms for the diagnosis/follow-up of rare diseases or education material developed by the laboratory). If necessary, additional on-sites visits were performed in March and April 2018 by Sciensano in order to assess practical aspects of the analysis’ validation and SOP.

### Reference laboratories recognition

**Fig. 4:**
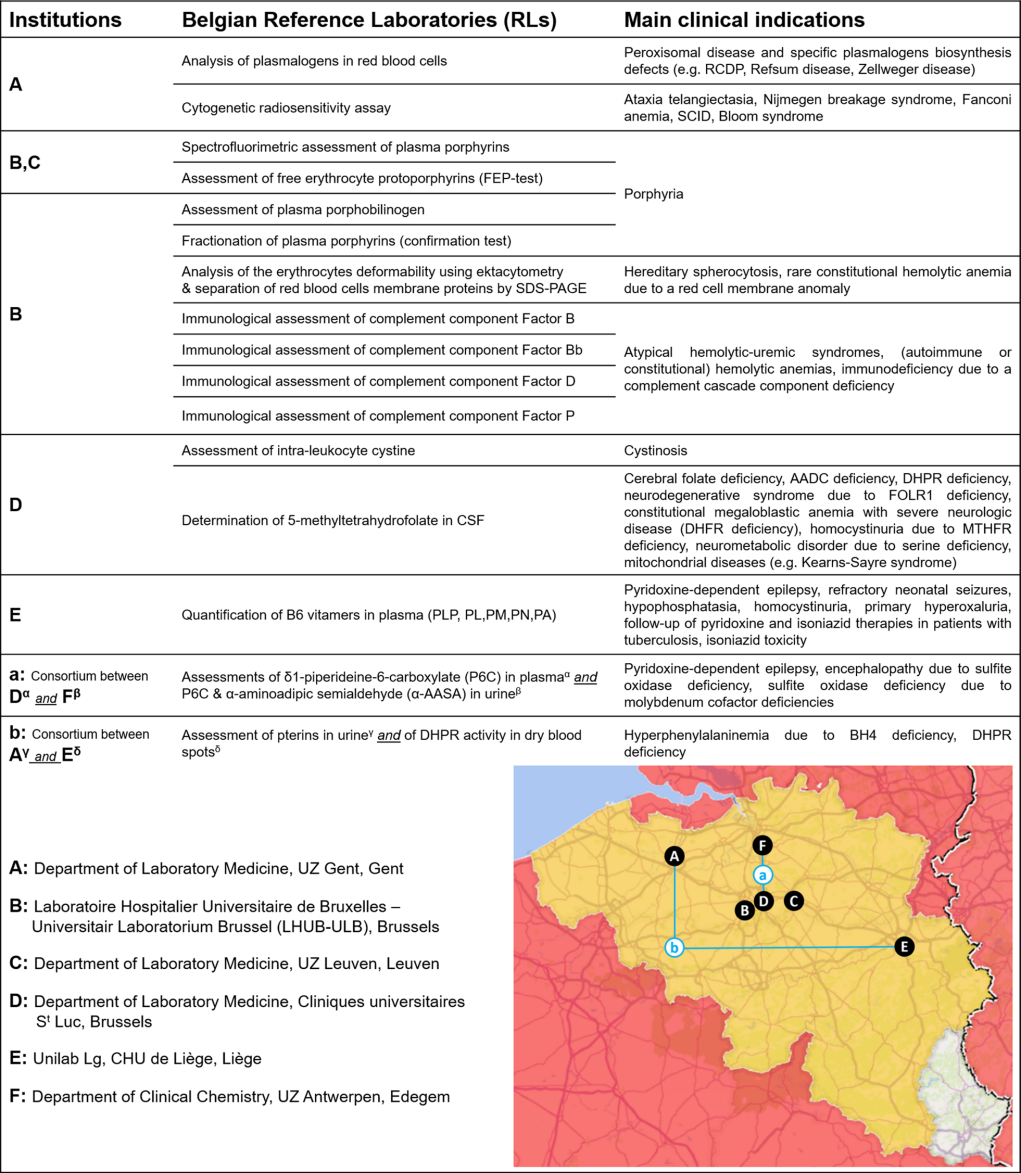
Representation of the new Belgian reference laboratories. *AADC* aromatic l-amino acid decarboxylase, *BH4* tetrahydrobiopterin, *CSF* cerebrospinal fluid, *DHFR* dihydrofolate reductase, *DHPR* dihydropteridine reductase, *FOLR1* folate receptor 1, *MTHFR* methylene tetrahydrofolate reductase, *PA* pyridoxic acid, *PL* pyridoxal, *PLP* pyridoxal-phosphate, *PM* pyridoxamine, *PN* pyridoxine, *RCDP* rhizomelic chondrodysplasia punctate, *SCID* severe combined immunodeficiency, *SDS-PAGE* sodium dodecyl sulfate polyacrylamide gel electrophoresis

Among the 18 analyses included in the call for application scope, RLs were recognized on 3 December 2019 for 16 analyses. The names of the institutions to which RLs belong, the names of the analyses for which they have been recognized and the main clinical indications of these tests, as well as RLs localization are illustrated in Fig. 4. All of them were Belgian university hospital laboratories which participate to External Quality Assessment schemes [EQAs] for 6 of the 16 analyses considered, international ring tests for 9 analyses, and a combination of both for 1 analysis). No applications were submitted for two analyses included in the call: assessment of the acid-labile subunit in serum and detection of the 14-3-3 protein in the cerebrospinal fluid.

RLs were set up in the form of a consortium of two laboratories for two analyses, namely for (1) the assessment of α-aminoadipic semialdehyde in urine and δ1-piperideine-6-carboxylate in plasma and urine (mainly used in the case of pyridoxine-dependent seizures), and (2) the assessment of pterins in urine and of the 6,7-dihydropteridine reductase activity in dry blood spots (used in the context of hyperphenylalaninemia).

For two other analyses (spectrofluorimetric assessment of plasma porphyrins and assessment of free erythrocyte protoporphyrins), two RLs have been recognized instead of one because of their similar quality, annual volume and long-term expertise recognized at the international level (membership to the European Porphyria Network for more than 10 years).

Prescription forms and criteria, as well as instructions for the sampling, storage and transport of the samples, have been developed for each RL.

The call for application for the Belgian RLs should be renewed every 5 years in order to allow modifications of the Belgian RLs scope based on adaptations of the activities of the Belgian laboratories of clinical pathology and tests’ availability in Belgium.

### Collaborations with foreign laboratories

**Fig. 5:**
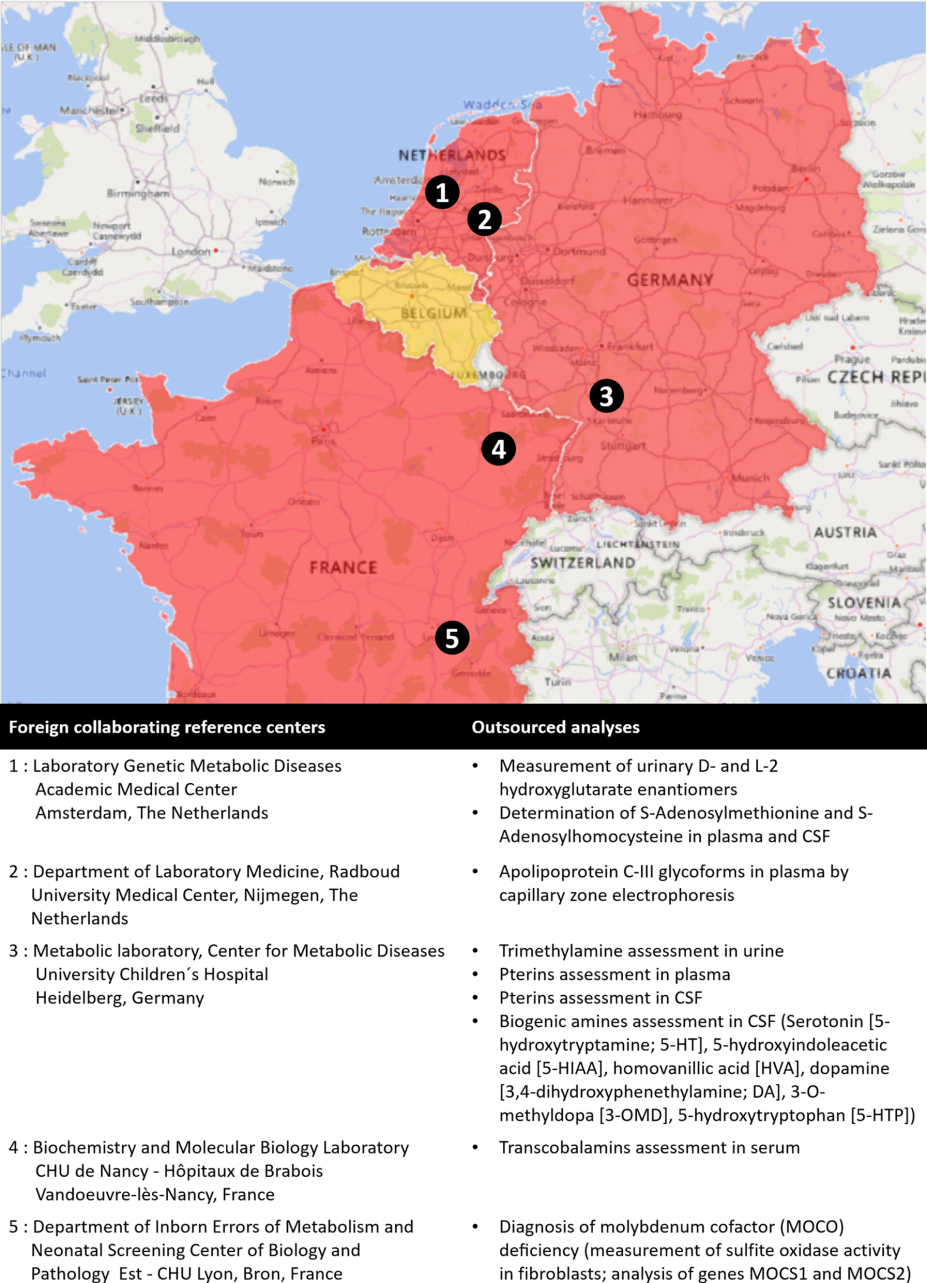
Foreign laboratories with whom collaborations were established for the outsourcing of analyses unavailable in Belgium. *CSF* cerebrospinal fluid

After the evaluation of the quality, expertise and costs of different European laboratories performing the priority analyses that are unavailable in Belgium, partnerships have been developed with 5 foreign partners for the outsourcing of 9 analyses (cf. Fig. 5). The efficiency, and quality of analyses outsourced abroad, as well as the needs of collaborations’ renewal based on the evolution of the tests availability in Belgium, will be annually reviewed by the RDWG.

### Impact of the project on the management of the patients with rare diseases

The implementation of new reimbursement conditions for a large number of analyses has improved the patients’ access to specialized diagnostic tests. Indeed, these analyses were previously performed for free by the Belgian medical laboratories or charged to the patients. Since the entry into force of the 42 new reimbursement codes, the laboratories are paid for the performance of these tests and the costs are no longer charged to the patients. Thus, between 1 April and 31 December, 2019, 8.599 tests could be reimbursed under the 42 new nomenclature codes. This represents a saving of €282.029,84 for the patients with rare diseases and Belgian medical laboratories in a nine-months period. The related expenditures of the INAMI-RIZIV had amounted to €70.507,46 (average: €7834,16 per month). The remainder (€211.522,38) had been charged to the health insurances of the patients.

Besides, the selection of RLs has mainly helped to improve the management of the patients with rare diseases by increasing the availability of some highly-specialized tests in Belgium and by establishing a control and follow-up framework for the RLs activities. In that respect, some analyses that were not available in Belgium before this study (i.e. the assessments of the dihydropteridine reductase activity in dried blood spots, B6 vitamers in plasma, pterins in urine, α-aminoadipic semialdehyde and δ1-piperideine-6-carboxylate in urine) have been developed by some Belgian university laboratories of clinical pathology after the presentation of the results of the feasibility study in 2016.

Concerning the quality of the tests, this project also enabled to rationalize the outsourcing and performance of rare analyses. Before 2016, some medical analyses covered by this project were heterogeneously outsourced to several Belgian or foreign laboratories, even if available in Belgium. This happened without any harmonization of the outsourcing procedure or possible control and follow-up of the volumes, quality, TAT, or costs of the tests by the Belgian healthcare authorities. In that respect, the recognition of Belgian RLs in 2019 enabled to centralize the performance of some analyses within one Belgian RL versus 5 (assessment of pterins in urine), 3 (assessment of 5-methyltetrahydrofolate in cerebrospinal fluid) or 2 (assessments of plasmalogen levels in erythrocytes, and α-aminoadipic semialdehyde and δ1-piperideine-6-carboxylate in urine) different medical laboratories in 2015. This has had a positive impact on patients’ management through the possible reduction of the TAT, and a better tracing of the samples and follow-up of the quality of the analyses.

Finally, the project also helped to reduce patients’ costs for analyses that are not available in Belgium. Since 1 January 2019, the shipment and analytical costs of the outsourced analyses are reimbursed through a specific financial envelop allocated by the INAMI-RIZIV.

### Impact of the project on the Belgian RLs’ cost-effectiveness

This project also had a positive impact on the Belgian RLs cost-effectiveness. Indeed, before the official RLs recognition on 3 December 2019, the costs of the medical analyses performed by these laboratories were not or only partially invoiced to the patients. This situation induced insufficient incomes for the laboratories that performed these tests. The RLs recognition and funding by the Belgian healthcare authorities helped to deal with this problem. The RLs are receiving a reimbursement for the analyses performed since the 1 January 2019. Moreover, since 2019, RLs’ costs related to their annual participation to quality controls and accreditation of the analyses for which they have been recognized are totally reimbursed. This is achieved through a specific annual envelop (€135.000 in 2019 and 2020) allocated by the INAMI-RIZIV.

**Fig. 6:**
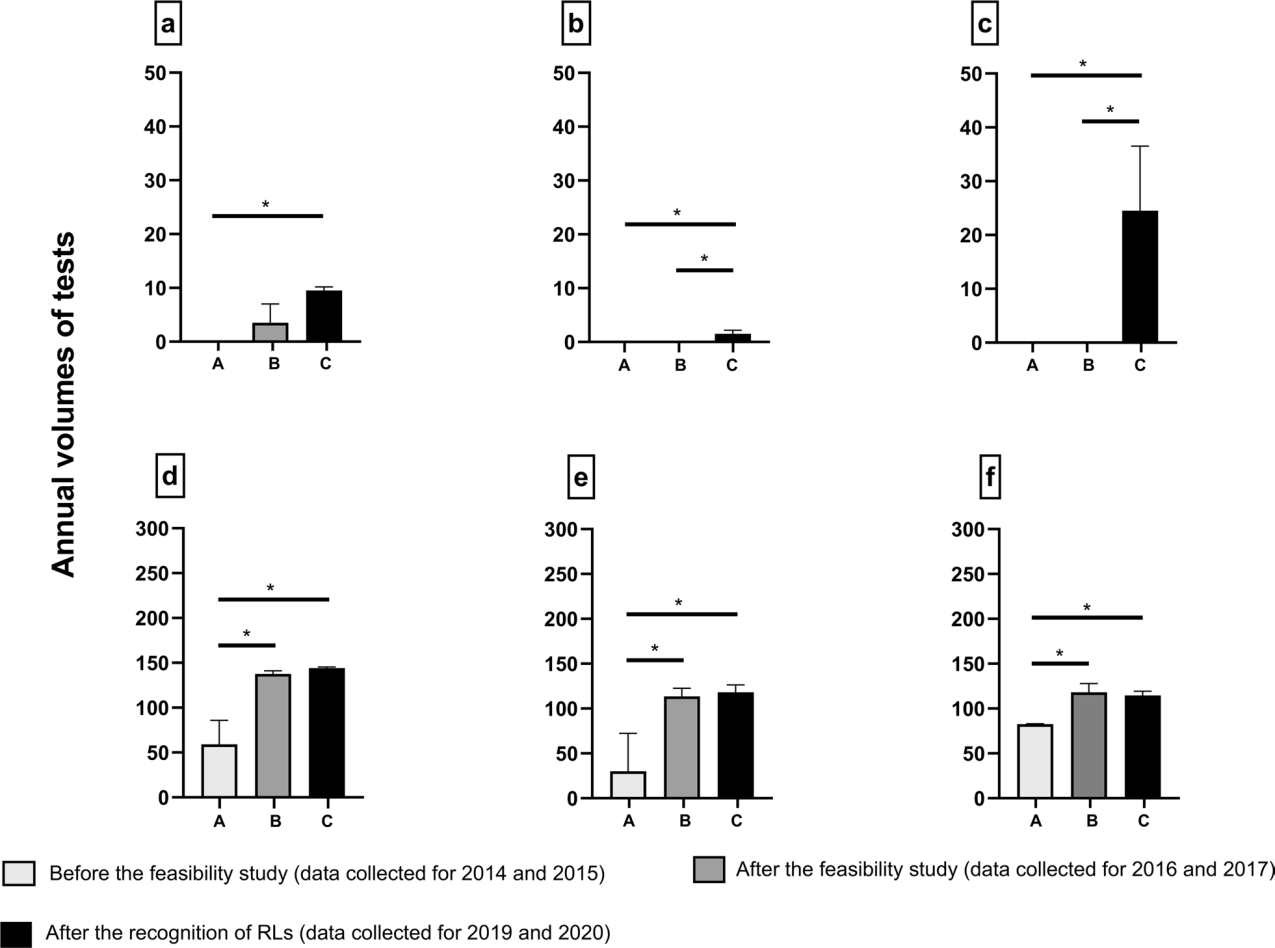
Significant impact of the feasibility study results on the annual volumes of six analyses. Comparison of the volumes of tests reported by the Belgian laboratories of clinical pathology for 6 different years: group A (light grey bars, period before the presentation of the results of the feasibility study [data collected for 2014 and 2015]) versus group B (dark grey bars, period after the presentation of the results of the feasibility study [data collected for 2016 and 2017]) versus group C (black bars, period from RLs’ recognition [data collected for 2019 and 2020]). Values were calculated as mean volumes ± SD, n = 2 for the 3 groups (A,B,C) of two successive years. Statistical analyses were performed by one-way ANOVA with Tukey’s posttest for multiple comparisons between the 3 groups. Asterisks indicate values that are statistically significantly different from each other (*p < 0.05). Analyses presented in each panel: **a** assessment of α-aminoadipic semialdehyde and δ1-piperideine-6-carboxylate in urine; **b** assessment of B6 vitamers in plasma; **c** assessment of pterins in urine; **d** assessment of Complement component Factor B; **e** assessment of Complement component Factor Bb; **f** assessment of intra-leukocyte cystine. *RLs* Reference laboratories

The proposal of centralization of some analyses within one Belgian RL formulated after the feasibility study (2016) also helped to increase the Belgian laboratories’ annual volumes of tests for those analyses, with a positive effect on the amortization of their equipment purchase costs, analyses’ validation costs and staff training costs. Figure 6 illustrates the comparison of the mean annual volumes collected for three different periods: before the presentation of the results of the feasibility study (group A, data collected for 2014 and 2015), after the presentation of the results of the feasibility study (group B, data collected for 2016 and 2017), and after the RLs’ recognition (group C, data collected for 2019 and 2020). Results are expressed as mean volumes ± SD for 2 successive years (n = 2). For the 6 analyses shown in this figure, a significant increase (*p* < 0.05) of the mean annual volumes of tests could be observed after the presentation of the results of the feasibility study in 2016. This highlights the positive impact of this project on the development of new analyses in Belgium (panels a–c) but also on the performance of some other analyses available in Belgium since many years (panels d–f). For the other analyses performed by RLs, no statistically significant modification of the annual volumes could be observed between 2014 and 2020 (see Additional file 1: Figure S1). This may be explained by the very low prevalence of the diseases for which these analyses are prescribed and therefore the limited number of tests that makes the inter-groups differences less important.

### Follow-up of costs and expertise recognition

An annual activity and financial report describing the activities, financing and renewal of the Belgian RLs of clinical pathology and the outsourcing of analyses to foreign laboratories will be annually sent to the INAMI-RIZIV.

## Discussion

This study is the first Belgian initiative for the improvement of the management of patients with rare diseases through the promotion and financing of analyses of clinical pathology.

Its approach derives from the “*RAND/UCLA appropriateness method*”, which aims to measure and validate the necessity and usefulness of clinical procedures based on the consensus of experts’ opinions [21, 22].

Several inputs of this project are worth mentioning. First of all, this study enabled to create a permanent working group composed of Belgian experts in rare diseases management within the Belgian Commission on Clinical Pathology. This will help to perform a continuous follow-up of rare diseases diagnosis and epidemiology in Belgium and of the efficiency and quality of medical analyses performed in these particular contexts. Moreover, the collaboration between this working group and Sciensano enabled the (1) identification of unmet financing needs for specific analyses of clinical pathology used for rare diseases’ diagnosis/follow-up, (2) set-up of coverage of these tests by the Belgian Healthcare system, under the surveillance of Sciensano and INAMI-RIZIV, and (3) establishment of reference laboratories (RLs) for the most specific tests.

The establishment by the Belgian healthcare authorities of a specific budgetary envelop dedicated to analyses of clinical pathology supports the usefulness of these tests in the context of rare diseases diagnosis and follow-up in parallel with genetic tests.

Nowadays, RLs have been recognized for a few types of medical analyses and in a limited number of countries. Most of them are focused on human genetics and microbiology tests [23–25]. The establishment of Belgian RLs for some analyses used in the context of rare diseases performed during this study echoes the previous recognition of Belgian National Reference Centers for human microbiology in 2011 [24] and is to our knowledge the first RL initiative in the context of rare diseases and for analyses of clinical chemistry, haematology and immunology.

The RLs recognition offers several advantages in terms of healthcare quality. First, it took place after a harmonized selection procedure for all tests considered. Second, affiliations of RLs to university hospitals improves the clinical management of the patients with rare diseases due to the close link between clinical and laboratory activities and reduced TAT. Third, RLs possess a highly-specialized equipment and perform the analyses using validated methods based on peer-reviewed publications and international guidelines. Fourth, RLs annually participate to external quality controls for the test(s) for which their expertise has been recognized. Fifth, the centralization of highly-specific tests to one or two RL(s) is cost-effective regarding the amortization of laboratory costs related to the performance of small tests’ annual volumes, as it is the case for rare diseases, and helps the prescribers to rapidly identify the RLs to which clinical samples have to be sent. Finally, RLs recognition and financing will also encourage the Belgian laboratories to develop and validate new tests that remain unavailable in Belgium.

In addition, the selection of RLs will have a positive impact on public health by offering useful expertise in rare diseases management to the medical authorities, healthcare professionals and patients and by helping to collect information about rare diseases in Belgium through national databases and registries.

Lastly, it is important to mention that recognition of RLs by the Belgian healthcare authorities gives the laboratory a particular visibility and notoriety at the national and international levels, and a responsibility in terms of healthcare excellence and scientific expertise. This helps RLs to expand their collaborations and to position themselves within the Rare Diseases European Reference Networks [26].

## Conclusions

This initiative of the Belgian plan for rare diseases (1) promotes healthcare quality and the expertise recognition and collaborations of Belgian rare diseases experts at the national and international levels, (2) offers a better financing to the laboratories of clinical pathology performing highly-specific analyses in the context of rare diseases, and (3) enables to reduces patients’ costs.

## Supplementary Information

**Additional file 1: Figure S1.** Analyses for which the mean annual volumes were not modified by the feasibility study results. Comparison of the volumes of tests reported by the Belgian laboratories of clinical pathology for 6 different years: group A (light grey bars, period before the presentation of the results of the feasibility study [data collected for 2014 and 2015]) versus group B (dark grey bars, period after the presentation of the results of the feasibility study [data collected for 2016 and 2017]) versus group C (black bars, period from RLs’ recognition [data collected for 2019 and 2020]). Values were calculated as mean volumes ± SD, n = 2 for the 3 groups (A,B,C) of two successive years. Statistical analyses were performed by one-way ANOVA with Tukey’s posttest for multiple comparisons between the 3 groups. The absence of asterisks indicates values that are not statistically significantly different from each other (*p* ≥ 0.05). Analyses presented in each panel: a: assessment of Complement component Factor D; b: assessment of Complement component Factor P; c: assessment of plasma porphobilinogen; d: fractionation of plasma porphyrins; e: assessment of the dihydropteridine reductase activity in dried blood spots; f: assessment of plasmalogen levels in red blood cells; g: cytogenetic radiosensitivity assay; h: assessment of δ1-piperideine-6-carboxylate in plasma; i: determination of 5-methyltetrahydrofolate in cerebrospinal fluid; j: analysis of the deformability of erythrocytes using osmotic gradient ektacytometry and separation of red blood cells membrane proteins by SDS-PAGE; k: assessment of free erythrocyte protoporphyrins; l: spectrofluorimetric assessment of plasma porphyrins. Abbreviations: RLs: Reference laboratories.

## Data Availability

The data management is coordinated by Sciensano, Belgium based on rules consented by the participating institutions. Interested research groups may apply for access and permission to analyze data, within the legal and ethical framework, through the Secure File Transfer Protocol (SFTP). Applications should be directed to the principal investigator: nathalie.vandevelde@sciensano.be.

## Abbreviations

BCCP: Belgian Commission on Clinical Pathology
EQA: External Quality Assessment
INAMI-RIZIV: National Institute for Health and Disability Insurance
RAND/UCLA: Research and Development/University of California, Los Angeles
RDWG: Rare Diseases Working Group
RL: Reference laboratory
SOP: Standard operating procedure
TAT: Turnaround time
